# EFFECTIVENESS OF FIXED DOSES OF PROPRANOLOL IN THE TREATMENT OF HEMANGIOMAS REGARDLESS OF CHILD’S WEIGHT GAIN: A CASE REPORT

**DOI:** 10.1590/1984-0462/;2019;37;3;00004

**Published:** 2019-05-09

**Authors:** Jaime Anger, Eduardo Mesquita de Oliveira, José Gabel

**Affiliations:** a Hospital Israelita Albert Einstein, São Paulo, SP, Brazil.; b Project “Einstein na Comunidade Paraisópolis”, São Paulo, SP, Brazil.

**Keywords:** Hemangioma, Propranolol, Rebound effect, Child, Hemangioma, Propranolol, Efeito rebote, Criança

## Abstract

**Objective:**

To present the outcomes of fixed doses of propranolol tablets for the treatment of hemangiomas.

**Case description:**

Two illustrative cases of hemangioma in infant patients younger than six months old are described. Treatments were started in 2010 and 2011 and were monitored until August 2017. Patients were treated with fixed doses, initially calculated based on the upper limit of 3 mg/kg/day and administrated in two daily doses rounded down to the nearest multiple of five milligrams. Dosage was not adjusted to patients’ weight gain. The tablets were crushed and then diluted in a maximum amount of 3 mL of water. This procedure was necessary because propranolol was not available in oral solution in 2009, when dosages available in the Brazilian market were 10, 40 and 80 mg. Both patients presented significative improvement in the first 60 days and were in complete remission by the end of the treatment.

**Comments:**

It is possible to treat patients with Propranolol 10 mg tablets, even though the dosage is not as precise as when calculated according to patients’ weight. The maintenance of a fixed dose, ignoring the patient’s progressive weight gains, helps avoiding the rebound effect and decreases complications.

## RESUMO

**Objetivo:**

Apresentar a experiência com a utilização de propranolol em doses fixas, em forma de comprimido, para o tratamento de hemangiomas.

**Descrição do caso:**

Dois casos ilustrativos de portadores de hemangiomas com menos de seis meses de idade são descritos. O início de tratamento ocorreu nos anos de 2010 e 2011 com seguimento até agosto de 2017. Os pacientes foram tratados com doses fixas iniciais calculadas com limite máximo de 3 mg/kg/dia, divididas em duas doses diárias, sempre com quantidades múltiplas de 5 mg. Os comprimidos de 10 mg ou a sua metade eram macerados e diluídos em 3 mL de água. As doses não foram mais alteradas. Esse uso foi decorrente da ausência da forma líquida de propranolol em 2009, quando começamos a utilizar esse tratamento, sendo então apenas disponíveis comprimidos de 10, 40 e 80 mg. Os pacientes obtiveram melhora acentuada nos primeiros 60 dias e remissão completa posteriormente.

**Comentários:**

É possível o uso de comprimidos de 10 mg, apesar de resultar numa dose não exata, como a calculada por kg/peso. A manutenção da mesma dose, mesmo com aumento progressivo de peso, pode evitar o efeito rebote e diminuir o índice de complicações.

## INTRODUCTION

Hemangiomas classified as childhood hemangiomas^1^ are vascular anomalies characterized by the proliferation of endothelial cells that arise after birth and increase progressively during the first year of life. In more than 95% of cases, children undergo a regression process, which can be fast or extend until the age of 10. However, even when small in size, they can cause complications.^1^ In 2008, oral treatment with propranolol was reported, with efficacy proven by multiple authors; currently, it is the initial treatment option.^2^

The dose of propranolol in the pioneer report of Léauté-Labrèze was 2 mg/kg/day divided into two doses to avoid complications. However, the same author, after an extensive review of cases in 2015,^3^ recommended 3 mg/kg/day for six months and mentioned the possibility of rebound effect after treatment discontinuation, which was also described by other researchers.^4^ ^,^ ^5^ Nevertheless, recent studies indicate that lower doses of up to 0.5 mg/kg/day may also be effective.^6^ ^,^ ^7^

At our institution, we have used propranolol as reference treatment in childhood hemangiomas since 2009, with initial dose of 3 mg/kg/day divided into two doses, but propranolol hydrochloride is only available as tablets of 10, 40 and 80 mg. In most children, treatment was started at less than six months of age and while they weighed less than 10 kg. Consequently, daily total dose calculation ranged from 15 to 20 mg of propranolol, leading to the use of 10 mg tablets in whole or divided into half, diluted in water. The tablet cannot be divided more than twice, with risk of dose error, and its contents, once fragmented and diluted in water, may not dissolve homogeneously in the solution, also leading to dose errors. For this reason, we started to use only multiple doses of 5 mg after being smashed and diluted in 3 mL of water. The final dose calculated should be always less than 3 mg/kg/day.

Most patients treated at our service showed the first signs of improvement after 40 to 60 days of treatment, which is in agreement with what has been described by several authors. In this period of improvement, the initial dose would still be calculated. The difficulty of continuously adjusting the dose to the children’s weight gain, as the medicine was being administered in tablets, allowed us to see a continuous improvement of their clinical picture, despite the stagnation and increase of dose discrepancy. This resulted in dose setting irrespective of weight gain. We could then notice that, even so, the doses varied according to the range of doses proposed by different authors at the period of their studies, that is, between 1 and 3 mg/kg/day.

In this article, we describe our experience by reporting the progress of two cases with complete treatment and total remission of hemangiomas.

## CASE DESCRIPTION

**Figure 1:**
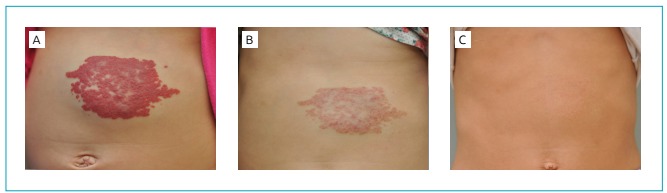
Patient 1: Abdominal region (A) before treatment; (B) at 60 days of treatment; (C) at six years of age.

Case 1 was a female patient born on February 10, 2011 weighing 4,600 g. She presented with a 2-cm red spot on the skin in the epigastrium region. At three weeks of life, the spot rapidly grew in extension, reaching an area of 13 x 8 cm (Figure 1A). After cardiac evaluation, treatment with propranolol at 138 days of age was started. The child weighed, at that time, 6,070 g. The dose administered was 10 mg in the morning and 5 mg in the evening, that is, 2.5 mg/kg/day. Hemangioma regression was seen at the second monitoring appointment, after 54 days of treatment (Figure 1B). The treatment was discontinued at 427 days of age and weight of 8,800 g, when the child received a dose of 1.7 mg/kg/day. Complete remission occurred around 4 years of age. No intercurrences related to treatment were seen (Figure 1C).

**Figure 2:**
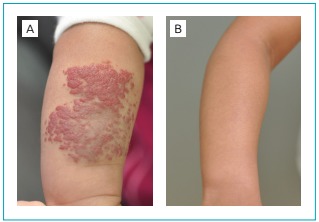
Patient 2: left forearm (A) on the first day of treatment; (B) at the age of 5, total hemangioma remission.

Case 2 was a female patient born on May 10, 2010 with 3,200 g of weight. The patient presented with a spot of rapid growth on the left forearm at two weeks of life. This spot size reached 8 x 5 cm at four weeks of age (Figure 2A). After cardiac evaluation, treatment was started at 128 days of life. At that time, the child weighed 5,060 g. The dose of propranolol administered was 10 mg in the morning and 5 mg in the evening, that is, 3.0 mg/kg/day. Improvement was first observed at the monitoring appointment after 42 days of treatment. Propranolol was discontinued at 397 days. At that time, the patient weighed 8,080 g and received 1.9 mg/kg/day. Complete remission was seen at 18 months of age. No treatment-related intercurrences were reported (Figure 2B).

## COMMENTS

The treatment of hemangiomas with oral beta-blockers was proven effective^8^ by multiple series of case reports,^8^ with propranolol hydrochloride as the leading option. The rate of complications is relatively low, and, for most children, it does not result in treatment discontinuation.^3^ ^,^ ^8^ ^,^ ^9^

Even after this recent experience, some topics are not yet clear, such as dose for treatment and how to avoid the rebound effect.^3^ There is no consensus on the ideal dose. Reports with larger groups of patients suggest 1 to 3 mg/kg/day. Some authors point to lower initial doses and increase until reaching the maintenance dose.^8^

When reviewing cases, we found that most patients were less than one year of life and their body weight varied from 5 to 7 kg, which allowed the use of a 10-mg tablet in whole or cut in half, divided into two daily doses. This means that the doses were not as accurate as intended, but effective, as seen in cases herein reported, in which doses remained in the range of 1.8 to 3.0 mg/kg/day.

Non-availability of liquid solution, which would allow a dose of 1 mg and consistent with calculated dose, resulted in forced maintenance of the same doses, since the increment of an entire tablet of 10 mg or its half (5 mg) would result in overdosage. Children’s weight gain and the relative decrease of doses coincided with the improvement in their clinical picture; one should note, however, that no patient received more than 3 mg/kg/day and in no case did treatment interruption happen with a dose lower than 1 mg/kg/day, probably avoiding the rebound effect after a sudden abrupt withdrawal of high doses, which had already been observed at our service when interferon was used to treat complicated hemangiomas.^10^

Therefore, propranolol can be administered in 10 mg tablets, although it results in non-exact dose, as calculated per kg of body weight per day. Maintaining the same dose, even with progressive weight gain of children as they grow, may avoid the rebound effect and should decrease the rate of complications. Starting and maintaining doses until the end of treatment simplifies care.
